# Haptic sound-localisation for use in cochlear implant and hearing-aid users

**DOI:** 10.1038/s41598-020-70379-2

**Published:** 2020-08-25

**Authors:** Mark D. Fletcher, Jana Zgheib

**Affiliations:** 1 University of Southampton Auditory Implant Service, University of Southampton, University Road, Southampton, SO17 1BJ UK; 2 Faculty of Engineering and Physical Sciences, University of Southampton, University Road, Southampton, SO17 1BJ UK

**Keywords:** Auditory system, Somatosensory system, Biomedical engineering, Translational research, Human behaviour

## Abstract

Users of hearing-assistive devices often struggle to locate and segregate sounds, which can make listening in schools, cafes, and busy workplaces extremely challenging. A recent study in unilaterally implanted CI users showed that sound-localisation was improved when the audio received by behind-the-ear devices was converted to haptic stimulation on each wrist. We built on this work, using a new signal-processing approach to improve localisation accuracy and increase generalisability to a wide range of stimuli. We aimed to: (1) improve haptic sound-localisation accuracy using a varied stimulus set and (2) assess whether accuracy improved with prolonged training. Thirty-two adults with normal touch perception were randomly assigned to an experimental or control group. The experimental group completed a 5-h training regime and the control group were not trained. Without training, haptic sound-localisation was substantially better than in previous work on haptic sound-localisation. It was also markedly better than sound-localisation by either unilaterally or bilaterally implanted CI users. After training, accuracy improved, becoming better than for sound-localisation by bilateral hearing-aid users. These findings suggest that a wrist-worn haptic device could be effective for improving spatial hearing for a range of hearing-impaired listeners.

## Introduction

Users of hearing-assistive devices, such as hearing aids and cochlear implants, often struggle to locate and segregate sounds^1–4^. As well as impairing threat detection, this makes listening in complex acoustic environments—such as, schools, cafes, and busy workplaces—highly challenging. Cochlear implants (CIs) enable severely-to-profoundly deaf individuals to perceive sound by electrically stimulating the auditory nerve. Recently, it has been shown that this electrical stimulation can be augmented by providing missing sound information through haptic stimulation (“electro-haptic stimulation”)^5–11^. Historically, a small number of studies in young normal-hearing listeners have explored the possibility of using haptic stimulation on the fingertips to locate sounds^12–15^, but research in this area is extremely sparse. In a recent study by Fletcher et al*.*, it was shown that sound-localisation can be substantially improved in CI users by augmenting the CI signal with haptic stimulation on the wrists^10^. This haptic stimulation was derived from audio signals that would be received by behind-the-ear hearing aids or CIs. Localisation accuracy increased after 30 min of training, with the same speech-sample used for both testing and training. In this study, we developed a new signal-processing strategy for haptic sound-localisation, which incorporated linked multi-band compression and wrist sensitivity correction. This was intended to improve haptic sound-localisation accuracy and increase the generalisability of the approach to a wide range of stimuli. The first aim of this study was to assess localisation accuracy with this new signal-processing strategy. To ensure that results were generalisable, accuracy was measured using a speech corpus that contained multiple talkers and sentences, with the talkers and sentence content used differing between testing and training sessions. The second aim was to assess whether haptic sound-localisation improved after a 5-h training regime.

For normal-hearing listeners, the dominant sound-localisation cues are time and intensity differences across the ears (interaural level and time differences; ILDs and ITDs). Unilaterally implanted CI users, who make up around 95% of the CI population^16^, often have little or no access to ILD and ITD cues. Implantation of a second CI can increase access to these cues, and thereby improve sound-localisation accuracy (although it should be noted that sensitivity often remains severely limited)^4,17–19^. This has led some researchers and clinicians to argue for much more widespread second implantation^20,21^. However, a second implant is expensive, risks vestibular dysfunction and the loss of residual acoustic-hearing, and limits access to future treatments and technologies. Haptic sound-localisation through stimulation on the wrists might offer an alternative that avoids the need for an expensive, invasive surgery to fit a second CI.

For the effectiveness of haptic sound-localisation to be properly assessed, it is important to establish the sound-localisation performance of CI and hearing-aid users. Localisation performance is typically reported as the root-mean-square (RMS) error in localisation. For the best performing CI users—those that are either bilaterally implanted or are unilaterally implanted with normal hearing in the non-implanted ear—RMS localisation error is ~ 28°^4^. This is similar to the 30° RMS error measured for sound-localisation using haptic stimulation in unilateral CI users by Fletcher et al*.*^10^. Note that, in these studies, performance was assessed using a single speech or noise stimulus, not a varied stimulus set. In Fletcher et al*.*, after training with the same single speech sample that was used for testing, RMS error for haptic sound-localisation was 26°. For bilateral hearing-aid users, average RMS error for sound localisation is ~ 25° for a varied stimulus set^22^. This compares to ~ 12° RMS localisation error for a single stimulus^4^ (although note that there are methodological differences between these studies and that different participants were used). In the current study, we set an ambitious target to match performance of bilateral hearing-aid users using a varied stimulus set.

For haptic stimulation across the fingers, the tactile system is highly sensitive to intensity differences, but not time differences^12,23,24^. It therefore seems likely that ILDs will be the dominant spatial-hearing cue for haptic sound-localisation on the wrists. Our signal-processing approach focused on maximising ILDs, which were delivered as intensity differences across the wrists. We also applied dynamic-range compression, which is used almost universally in hearing-assistive devices to compensate for the reduced dynamic-range available to the user^25^. Equal gain was applied to each wrist by the compressor to prevent distortion of ILD cues^26^. Dynamic-range compression was applied independently for different frequency bands, which reduced differences in the distribution of sound energy across frequency for different stimuli. ILDs are frequency dependent, due to acoustic head-shadowing^27^, and stimuli with different frequency content can therefore yield a different overall ILD for the same location. By equalising the contribution of each frequency band, the multi-band compression approach used in the current study aimed to reduce this variance in correspondence between the overall stimulus ILD and location. In addition to applying linked multi-band compression, for each individual, the overall intensity of haptic stimulation at each wrist was adjusted to account for differences in sensitivity. This was intended to make the task more intuitive, both by ensuring that the 0° position felt equal on each wrist, and that the intensity difference between the wrists felt equally offset for left and right locations.

In the current study, we assessed the effectiveness of training for improving participants’ ability to locate sounds using only haptic stimulation. Several studies have shown the importance of training to exploiting auditory information presented through haptic stimulation. Fletcher et al*.* showed that a short training regime can improve sound-localisation accuracy for unilaterally implanted CI users both with and without a hearing-aid in the other ear, either when using audio alone, haptic stimulation alone, or a combination of audio and haptic stimulation^10^. This finding is supported by other work showing that sound localisation improves with training for CI users using a single CI^10,28^ and for individuals with a severe-to-profound unilateral hearing loss^29^. Another set of studies have shown that training is important for CI users to make effective use of haptic stimulation to improve speech-in-noise performance^5,6,11^. Previous researchers have also attempted to provide speech information through haptic stimulation as a substitute, rather than a supplement, to auditory stimulation. This work suggests that participants continue to improve their ability to extract speech information from haptic stimulation after many hours of training^30–32^.

In this study, we measured haptic sound-localisation accuracy before and after a 5-h training regime, consisting of ten training sessions that were each completed on separate days. Thirty-two adults with normal touch perception were randomly assigned to either an experimental or control group. In testing and training sessions, the participant’s task was to locate male or female speech stimuli, with different talkers and sentences used across sessions and no material repeated. For the experimental group, we set a target of achieving less than 28° RMS localisation error before training, as we expected our use of linked multi-band compression and wrist-sensitivity correction to give immediate benefit. This would mean we had substantially exceeded the performance achieved by Fletcher et al*.* before training for haptic sound-localisation with a single stimulus^10^. It would also mean we had exceeded sound-localisation performance in bilateral CI users for a single stimulus^4^. After training, we set another ambitious target of achieving haptic sound-localisation accuracy of 25° RMS error. This would exceed the haptic sound-localisation accuracy achieved after training by Fletcher et al*.* for a single stimulus^10^ and match the sound-localisation accuracy achieved by bilateral hearing-aid users for a varied stimulus set^22^.

## Results

**Figure 1:**
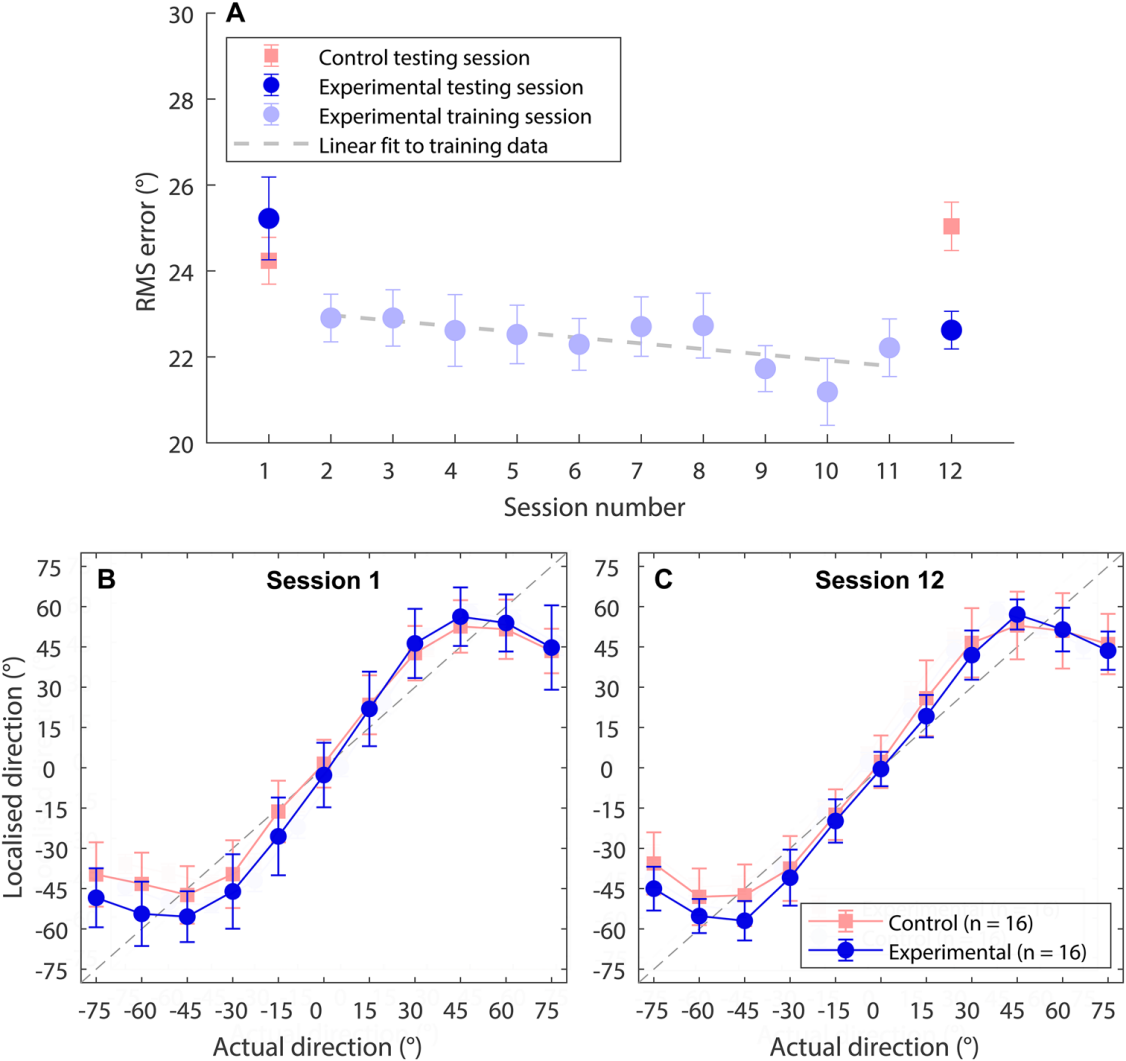
(**A**) Root-mean-square (RMS) localisation error for each session, for the experimental (light and dark blue circles) and control (light red squares) groups, each of which contained 16 participants. A linear fit to the training data is also shown (grey dashed line). Panel (**A**) error bars show the standard error of the mean. (**B**, **C**) Mean response location vs actual sound source location in the first (**B**) and final (**C**) testing sessions (the grey dashed-line shows perfect localisation performance). Panel (**B**, **C**) error bars show the standard deviation.

Panel A of Fig. 1 shows RMS haptic-sound localisation error for each session for both the experimental and control groups. Panels B and C show where participants perceived the stimulus to originate from compared to its true location, before and after the training period. Haptic stimuli came from one of eleven equally spaced locations, from 75° to the left and right of centre. These positions were displayed in an arc around the participants. Participants were instructed to identify the position the stimulus originated from.

We found no overall effect of testing session (before or after training; *F*(1,30) = 2.55; *p* = 0.121) or group (experimental or control; *F*(1,30) = 0.93; p = 0.343) on haptic sound-localisation accuracy. However, we did find a significant interaction between session and group (*F*(1,30) = 9.12; *p* = 0.005), indicating that the effect of session differed between the experimental and control groups. A statistically significant reduction in RMS localisation error was found in the second testing session for the experimental group (*t*(15) = 2.53, *p* = 0.046) but not for the control group (*t*(15) = -1.74, *p* = 0.103). For the experimental group, the RMS localisation error reduced by 2.6° after training, from 25.2° to 22.6°, with this effect ranging across participants from a reduction in error of 12.7° to an increase in error of 3°. Before training, the RMS localisation error ranged from 19.7° to 37.6° and, after training, from 20.0° to 25.9°. For the control group, the RMS localisation error was 24.2° in the initial testing session and 25.0° in the final testing session, ranging from 21.3° to 27.6° in the initial session and from 20.9° to 29.2° in the final session. For the experimental group, a Pearson’s correlation revealed that those who performed more poorly before training tended to improve most after training (*r* = 0.91, *p* ≤ 0.001).

A linear fit (using the least-squares method) was applied to the training data, as there was substantial variance both within and across sessions. It should be noted that feedback was provided during training, which may have enhanced performance estimates for the training sessions. The estimated improvement in performance across sessions was 1.2°, with performance reducing from 23.0° to 21.8°. The worst performer in any training session was 30.2° (2nd training session) and the best was 16.0° (9th training session).

**Figure 2:**
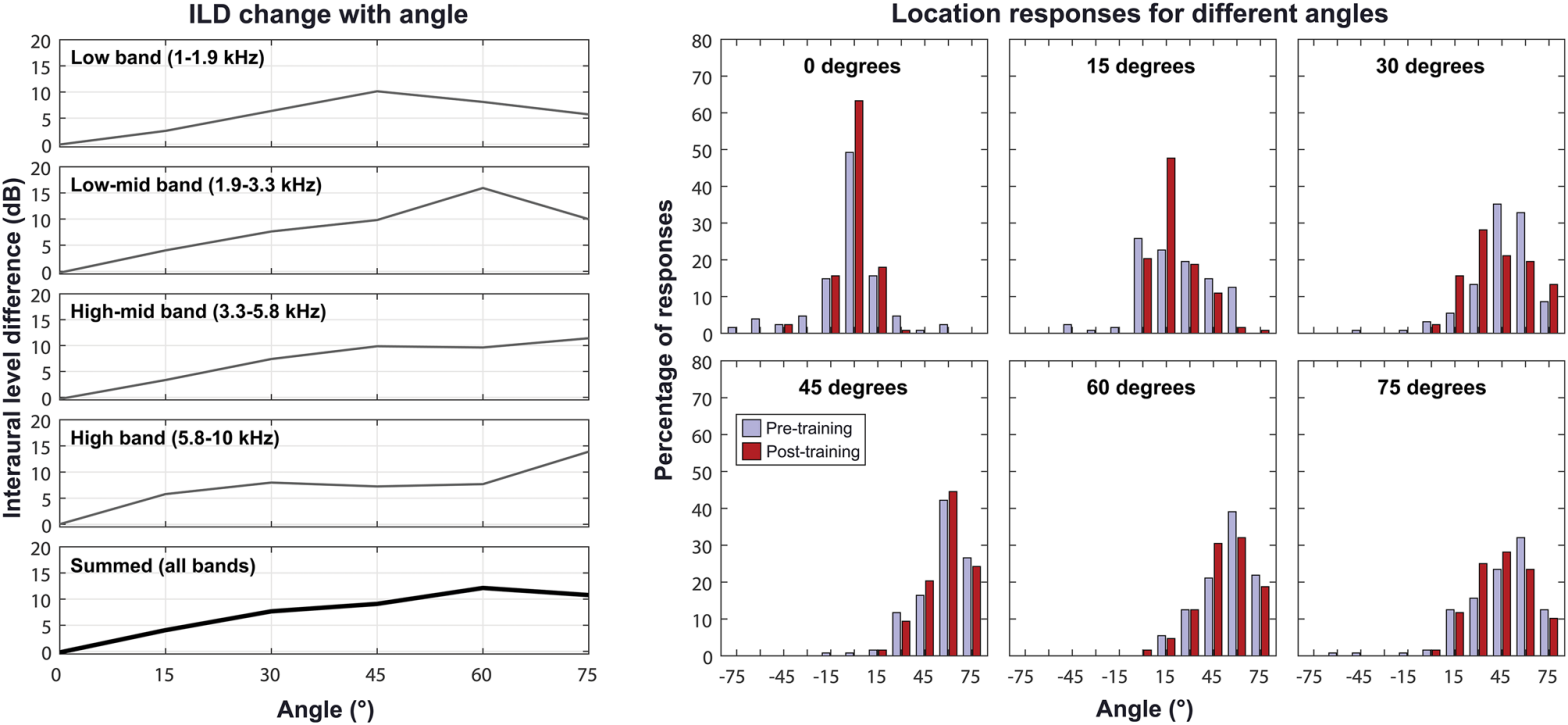
Left: Interaural level differences (ILDs) as a function of angle for haptic stimulation in the current study. ILDs for each frequency band used in the experiment and for the sum of all bands is shown for a speech-shaped noise stimulus. Right: Percentage of responses at each position for each sound location used in the experiment. Results are shown both before (light blue bars) and after (dark red bars) training for the experimental group. Data from locations on the left side have been combined with data from the right side (as the left and right responses were symmetrical).

Further analysis was conducted to explore how intensity differences across the wrists and response profiles changed with source location. Figure 2 (left) shows how the ILD changed with angle for haptic stimulation in the current study. ILDs are shown for a noise stimulus, with a frequency-spectrum matching the average spectrum of the BKB male talker sentences used in the testing sessions. When summed across all bands, the ILD change with angle was non-monotonic, increasing with increasing angle between 0° and 60° and decreasing with increasing angle between 60° and 75°. Furthermore, the variation in ILDs across frequency bands increased substantially for more lateral locations. At 15°, 30° and 45° locations, the maximum difference in ILD between bands was 3.2 dB, 1.6 dB, and 2.9 dB, respectively. In contrast, at both 60° and 75°, the maximum difference was 8.2 dB. Correspondingly, for angles less than 45°, haptic sound-localisation performance for the experimental group improved by 4.8° RMS error with training (from 23.2° to 18.5°; *t*(15) = 3.11, *p* = 0.029) but, for angles greater than 45°, no change in performance was found (mean increase in error of 1.1° RMS error, from 27.0° to 28.1°; *t*(15) = − 0.45, *p* ≥ 0.999). For the control group, performance was unchanged either for angles less than 45° (mean increase in RMS error of 1.2°, from 19.3° to 20.5°; *t*(15) = − 1.80, *p* = 0.276) or greater than 45° (mean increase in RMS error of 0.2°, from 30.1° to 30.4°; *t*(15) = − 0.33, *p* ≥ 0.999).

Figure 2 (right) shows the distribution of responses for each location before and after training for the experimental group. For source locations of 0°, 15°, and 30°, the number of correct responses increased substantially with training (from 49.2 to 63.3% at 0°, from 22.7 to 47.7% at 15°, and from 13.3 to 28.1% at 30°). The number of responses within 15° of the correct location (the next nearest possible source location) also increased appreciably (from 79.7 to 96.9% for 0°, from 68.0 to 86.7% for 15°, and from 53.9 to 64.8% at 30°). For a source at 30°, however, a clear bias towards more lateral positions, that was reduced by training, was apparent. Before training, the majority of responses were at 45° (35% compared to 13.3% at 30°) and after training the majority of responses were at 30° (28.1% compared to 21.1% at 45°). For the 45° source location, more modest improvements were seen with training and there was substantial confusion with the 60° location. Notably, before training, 42.2% of responses were at 60° while 16.4% were at 45° and, after training, 44.5% were at 60° while 20.3% were at 45°. For the 60° source location, training appeared to increase confusion between the 45° and 60° positions. After training, the number of 60° responses reduced from 39.1 down to 32.0% and the number of 45° responses increased from 21.1 to 30.5%. Finally, for the 75° position, there was a substantial bias towards less lateral locations, which increased with training. After training, responses of 45° increased from 23.4 to 28.1% and responses of 30° from 15.6 to 25.0%.

## Discussion

Haptic sound-localisation accuracy before training was substantially better than expected, with an average RMS localisation error of just 25.2° for the experimental group. This is markedly better than sound-localisation accuracy for CI users implanted in one ear—either with (~ 62° RMS error) or without (performing at chance) a hearing aid in the other ear—and for those with normal hearing in one ear and profound deafness in the other (~ 62° RMS error)^4^. Remarkably, haptic sound-localisation performance before training was also substantially better than that achieved by CI users implanted in both ears (~ 29° RMS error), users with a CI in one ear and healthy hearing in the other (~ 28° RMS error), and those with a CI in one ear and preserved low-frequency acoustic hearing in the other (~ 30° RMS error)^4^. Furthermore, these studies all assessed localisation performance using a single repeated stimulus, rather than a varied stimulus set as in the current study. Haptic sound-localisation accuracy before training was also substantially better than the 30° RMS error in haptic sound-localisation measured before training by Fletcher et al*.*^10^, who also used a single repeated stimulus. However, it should be noted that the average age of participants in this previous work was 53, compared to 26 in the current study. While vibrotactile detection thresholds are known to increase with age^33–35^, no effect of age on sensitivity to intensity differences has been found^36^. Haptic sound-localisation, which likely relies on exploitation of ILD cues, might therefore be expected to be robust to aging. An alternative reason that performance before training may have been improved compared to Fletcher et al*.* is the novel use of linked multi-band compression and wrist sensitivity correction in the current study.

Haptic sound-localisation was found to improve after 5 h of training (using a different speech corpus to that used in testing), reducing from 25.2° to 22.6° RMS error. Those performing worst before training were found to improve most with training. Accuracy after training was substantially better than haptic sound-localisation performance measured after training in Fletcher et al*.*^10^ (26° RMS error), in which participants were tested using the same speech sample that was used in training. In fact, the worst performer after training in the current study performed with the same accuracy as the average participant in Fletcher et al*.* Strikingly, the accuracy measured after training in the current study was also better than the average sound-localisation performance of bilateral hearing-aid users (~ 25° RMS error using a varied stimulus set)^22^. Overall, the current findings suggest that haptic stimulation can be used to locate sounds with an accuracy that would be highly beneficial to a large portion of hearing-assistive device users. Furthermore, they indicate that this approach can be effective across a range of speech samples, which is critical for its successful use in a real-world application.

The reduction in haptic sound-localisation error of 2.6° after 5 h of training was more modest than measured in Fletcher et al*.*^10^, where a reduction of 4.4° was found after just 30 min of training. The training approach used was the same in each study. There are two key differences between the studies that may explain the differences in improvement with training. Firstly, participants performed worse before training in Fletcher et al*.*, which may have been due both to differences in the signal-processing strategies used and the higher average age of participants in Fletcher et al. It may be more difficult to obtain benefit when localisation error is lower initially. Secondly, in Fletcher et al., the same single speech sample was used for training and testing. This may have resulted in stimulus-specific learning, which might not generalise to a broader stimulus set. It is highly encouraging that, in the current study, the benefits of training with a different speech corpus were successfully transferred to the testing session. This suggests that learning was not stimulus specific and is therefore likely to generalise to a broader range of stimuli. Generalisability across stimuli will be critical to the effectiveness of this approach in a real-world application.

Performance appeared to improve gradually with training, although there was high variance both within and across sessions. This variance was likely due to the fact that speech samples were drawn at random from a large and diverse corpus. It appears that the chief limit on performance was confusions at more lateral locations, where ILDs varied most across frequency (see Fig. 2). Each speech sample contains a different distribution of energy across frequency bands, which could lead to differing overall ILDs for a given location. This may have led to increased localisation error across sentences. However, as discussed in the Introduction, this effect will likely have been substantially reduced by the use of linked multi-band compression.

If haptic sound-localisation is to be effective for use with hearing-assistive devices, it is important that haptic and audio information is effectively combined in the brain. There is a range of evidence supporting the idea that audio and haptic information are effectively integrated. One previous study with CI users has shown that audio and haptic information can be effectively combined to improve sound localisation, after a short training regime^10^. Other studies have also shown that, with training, haptic information can be effectively combined with audio information to improve speech-in-noise performance^5,6,37,11^. There is a range of other behavioural evidence that audio and haptic information is effectively combined. For example, it has been shown that haptic stimulation can facilitate detection of quiet sounds^38^ and can modulate syllable perception^39^ and perceived loudness^40^. Extensive links between the auditory and tactile systems have also been found in anatomical and psychological studies, with extensive connections between the systems at both early and late processing stages^41–47^.

In future work, it may be possible to extend the signal-processing strategy used in the current study. One way in which the current strategy might be improved is through further enhancement of level difference cues (e.g., Francart et al.^48^). Particular focus might be given to improving performance at more lateral locations, where the most substantial confusions occurred. Another area that should be explored is whether the current signal-processing strategy can be used to enhance sound segregation in complex acoustic scenes, containing several concurrent sounds at different locations. Previous studies have shown that haptic stimulation can be used to enhance speech-in-noise performance for co-located speech and noise both for CI users^5,8^ and normal-hearing listeners listening to CI simulations^6,9^. Recently, using a similar approach to the current study, large enhancements have also been shown in speech recognition for a spatially separated speech and noise source^11^. However, due to the limited frequency information that can be provided with the current approach, it may be difficult to separate several simultaneous sound sources. This issue might be partly mitigated by mapping pitch or frequency to location on the skin, as in haptic devices such as the moasicOne_B^7^ and Tactaid VII (Audiological Engineering Corp). This approach might also allow participants to more effectively exploit across-band ILD cues, particularly at more lateral locations. Two previous studies of haptic enhancement of speech-in-noise performance have shown that an expander (which, in contrast to a compressor, expands the dynamic range of the signal) can be used as an effective noise-reduction strategy for speech in noise for sources coming from a single location^5,6^. Future studies could explore the effectiveness of using a linked expander for enhancing speech recognition in spatially separated noise.

In the current study, even without training, participants with normal touch perception were able to localise sounds using haptic stimulation on the wrists with an accuracy that was substantially better than CI users. This was achieved using a varied stimulus set and so is likely to be more representative of real-world performance than synthetic sounds or single audio samples used in many studies of spatial hearing. Remarkably, after training, performance was better than that of bilateral hearing-aid users. Our approach could therefore be highly effective for improving spatial hearing for a range of hearing-impaired listeners. Furthermore, the approach is designed to be suitable for use in a real-world application: haptic stimulation was delivered to the wrists, where devices are already routinely worn, and our new signal-processing strategy could readily be implemented in real-time on a compact, low-powered device. This device could offer a low-cost means of improving sound localisation across a broad range of hearing-assistive device users. Furthermore, it could offer a non-invasive alternative to fitting a second CI, while offering substantially greater improvements in sound-localisation accuracy.

## Methods

### Participants

Thirty-two adults with normal touch perception were recruited from the staff and students of the University of Southampton, and from acquaintances of the researchers. Participants were randomly assigned to either the experimental group (7 males, 9 females, aged between 21 and 31 years, with an average age of 25) or control group (6 males, 10 females; aged between 21 and 32 years, with an average age of 25). Participants were not informed of the aims of the study or which group they had been assigned to until the study was complete. Participants gave informed consent to take part and were paid £10 per hour. All participants reported no touch perception issues and had vibrotactile detection thresholds within the normal range (see Procedure).

### Stimuli

The experiment included both testing and training phases (with the control group only completing testing phases). The first testing session included intensity matching across the wrists. Intensity matching was performed using a randomly selected male (ID-01; list 3, sentence 2) and female (ID-07; list 2, sentence 3) sentence from the ARU British English speaker corpus of IEEE sentences^49^. Haptic sound-localisation measurements in the testing phases was performed with the male and female talker from the Bamford–Kowal–Bench (BKB) British English sentence corpus^50^. Haptic sound-localisation measurements for the training phases were performed with two male (ID-06 and ID-10) and two female (ID-08 and ID-09) talkers from the ARU corpus (who differed from those used for intensity matching). The talkers and sentence text differed between the testing and training sessions and, for each participant, no sentence was repeated at any stage of the experiment.

For all sentences, the intensity was normalised following ITU P.56 method B^51^. The intensity of the vibration stimulus was nominally set to 1.84 ms^−2^ RMS (frequency-weighted according to the weighting defined in BS-6842:1987^52^) for the position with maximum intensity (e.g. the left vibrometer when the signal was presented 75° to the left), as in Fletcher, et al.^10^. This level was roved by ± 2.5 dB (with a uniform distribution) to prevent participants using absolute level cues for localisation. The intensity at each wrist was adjusted for each individual so that the intensity felt equal in the 0° position (see Procedure). The average difference between the two wrists in the experimental group was 1.3 dB [max = 4.1 dB; min = 0.1 dB; standard deviation (SD) = 0.9 dB] and for the control group was 1.9 dB (max = 5.3 dB; min = 0.2 dB; SD = 1.5). All stimuli had total harmonic distortion of less than 0.1%. For all measurements, white noise was delivered to both ears at a level of 55 dB SPL to mask any audio cues from the shakers.

### Signal processing

**Figure 3:**
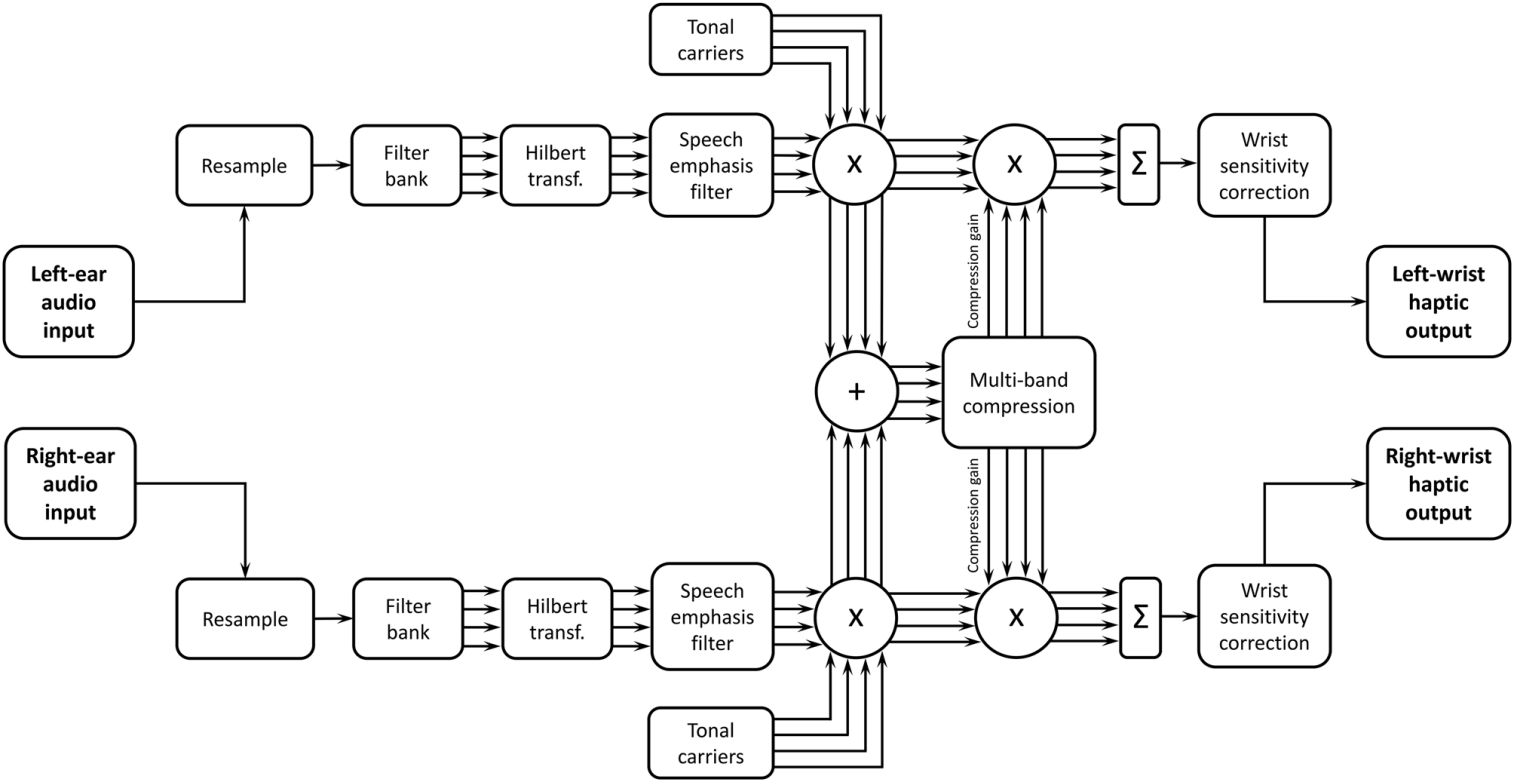
Schematic representation of the signal-processing chain.

Figure 3 shows each stage of the signal-processing approach used. The first stages matched that used in Fletcher, et al.^10^. Audio was first convolved with a head-related transfer function (HRTF) for the appropriate sound source location. The three-microphone behind-the-ear (“BTE_MultiCh”) HRTFs from The Oldenburg Hearing Device HRTF Database^53^ were used, in order to match a typical CI or hearing-aid signal. The signal from left and right ear channels were then resampled to a sampling frequency of 22,050 Hz. Each channel was then passed through an FIR filter bank with four frequency channels, which were equally spaced on the ERB scale^54^. The lower and upper edges of the bands were between 1,000 and 10,000 Hz. This frequency range was selected to contain both significant speech energy^55^ and large ILDs^27^. Next, a Hilbert transform was applied for each frequency channel to extract the amplitude envelope. A first-order low-pass filter was then applied with a cut-off frequency of 10 Hz to emphasize frequencies that are most important for speech intelligibility^56^. These four envelopes were then used to modulate the amplitudes of four fixed-phase tonal carriers with centre frequencies of 50, 110, 170, and 230 Hz. This frequency range was selected because it is one in which the tactile system is highly sensitive^57^. These carrier frequencies were also expected to be individually discriminable based on estimates of vibro-tactile frequency difference limens^58^.

Next, two additional signal-processing stages were conducted that were not used by Fletcher et al.^10^. First, linked multi-band dynamic-range compression was applied. Compression gains were calculated from the sum of the left and right channels for each carrier tone. Often, an alternative approach is used in which the maximum compression calculated for compressors at either ear is applied to both ears^26^. Here, compression based on the sum of both ears was preferred so that overall intensity information could be better maintained. There is also evidence from a study which used a related approach that this summing before compression might create narrower, less defuse sound images^59^. The dynamic-range peak compressor MATLAB system object (MATLAB R2019b, The MathWorks Inc., Natick, MA, USA) was used with the same parameters applied for each carrier. The threshold was set to − 40 dB, the compression ratio was 3:1, and the attack and release times were 10 ms and 20 ms. These parameters were selected to equalise the energy across the carriers while minimizing distortion of the amplitude envelope. The compression gain for each carrier tone was then applied equally to the left and right channels. This linked-compression approach was used to avoid distortion of ILD cues^26^. Following this stage, the carrier tones were summed for each channel. Finally, a further gain correction was applied to the left and right channels to correct for individual differences in sensitivity across the wrists. The signals were then delivered to the left and right wrists using two *HVLab* tactile vibrometers.

### Apparatus

During both testing and training sessions, participants sat in a quiet room. In front of them were left and right tactile vibrometers, placed a shoulder’s width apart, and a 24-inch monitor. Participants were encircled by a plastic ring with 11 labels equally spaced in an arc from 75° to the left and right (centred on the monitor) to match the locations used for the experiment.

All stimuli were controlled using a custom MATLAB script (version R2019b, The MathWorks Inc., Natick, MA, USA). Both audio (used to mask any sound from the shakers) and haptic signals were played out via an RME Babyface Pro soundcard (Haimhousen, Germany; sample rate of 48 kHz and bit depth of 24 bits). Audio was presented using ER-5A insert earphones (Etymotic, IL, USA). Two *HVLab* tactile vibrometers were placed on a foam surface in front of the participant to deliver the haptic stimulation to the participants’ wrists (the palmer surface of the distal forearm). The vibrometers were adapted by the substitution of the standard 6-mm probe with a 10-mm probe and the removal of the rigid surround. These changes increased the area of skin excitation. During screening, vibro-tactile threshold measurements were made following International Organization for Standardization specifications^60^. For these measurements, a *HVLab* Vibro-tactile Perception Meter was used with a 6-mm contactor, a rigid surround, and a constant upward force of 2 N.

All vibrometers were calibrated using a Bruel and Kjaer (B&K) type 4294 calibration exciter and PCB Piezotronics ICP 353B43 accelerometers. Audio stimuli were calibrated using a B&K G4 sound level meter, with a B&K 4157 occluded ear coupler (Royston, Hertfordshire, UK). Sound level meter calibration checks were carried out using a B&K Type 4231 sound calibrator.

### Procedure

The experiment consisted of two testing sessions, separated by 17 days. The first session began with screening and intensity-matching phases, followed by a break of at least 15 min. The first testing session lasted around one hour, and the final testing session lasted around 25 min. Between the two testing sessions, participants in the experimental group completed 10 training sessions on separate days. The maximum gap permitted between training sessions was two days. Each training session lasted around 30 min.

In the screening phase, participants first completed a questionnaire to ensure that they (1) had no conditions or injuries that might affect their touch perception, (2) had not been exposed to sustained periods of intense hand or arm vibration at any time, and (3) had no recent exposure to intense hand or arm vibration. Following this, participants had their vibro-tactile detection thresholds measured at the fingertip of the left and right index fingers to check for normal touch perception. Thresholds were measured following International Organization for Standardization specifications^60^. Participants were required to have touch perception thresholds in the normal range (< 0.4 ms^−2^ RMS at 31.5 Hz, and < 0.7 ms^−2^ RMS at 125 Hz^60^). Finally, otoscopy was performed to ensure insert earphones could be safely inserted. If the participant passed all screening stages, they moved to the intensity-matching phase.

In the intensity-matching phase, participants completed eight intensity matches. The same signal processing was used as in the testing and training sessions for the 0° location. The participant’s task was to adjust a slider until the left and right wrists felt equal in intensity. For half of the trials, the participants adjusted the intensity of the left wrist, and the other half they adjusted the intensity of the right wrist. The starting intensity of the adjusted wrist was nominally set to 2.5 dB above the intensity of the non-adjusted wrist for half of the trials, and 2.5 dB below for the other half. This starting level was roved by ± 1 dB (with a uniform distribution). Trials were repeated for both male and female speech samples. All intensity matches were averaged to calculate the intensity difference to be used in the testing and training sessions. Participants then moved to the first testing phase.

For both testing and training sessions, participants completed 88 trials, 44 of which used male and 44 of which used female sentences. The participant’s task was to identify which of the 11 possible stimulus locations (− 75° to + 75°) the haptic stimulus originated from. Responses were made verbally and recorded by the experimenter, who was blinded to the correct location. Each stimulus location was repeated 8 times, with the order of trials randomised. Localisation accuracy was calculated as RMS error using the *D* statistic described by Rakerd and Hartmann^61^. In training sessions, performance feedback was provided on the computer monitor in front of the participant. The screen displayed an illustration of the 11 stimulus locations, marked by boxes in an arc around an image of a person. If the participant was correct, the box at the correct location lit up green for 1 s. If the participant was incorrect, the box at the location selected lit up red for 1 s, and then the box at the correct location lit up green for 1 s. In the testing sessions, no feedback was provided.

The experimental protocol was approved by the University of Southampton Faculty of Engineering and Physical Sciences Ethics Committee (ERGO ID: 47769). All research was performed in accordance with the relevant guidelines and regulations.

### Statistics

Haptic sound-localisation accuracy was calculated as the RMS error from the target location in degrees arc for all trials within a session^61^. Levene’s and Kolmogorov–Smirnov Tests showed homogeneity of variance and revealed no deviation from normality. Primary analysis consisted of a mixed-ANOVA with within-subject factor “Session” (first or last) and between-subject factor “Group” (experimental or control). Post-hoc two-tailed paired-samples *t*-tests (with Bonferroni-Holm correction for multiple comparisons) were used to investigate these effects. A Pearson’s correlation was also performed to assess whether performance before training predicted the amount of benefit from training.

## Data Availability

The dataset from the current study is publicly available through the University of Southampton’s Research Data Management Repository at: 10.5258/SOTON/D1489.
